# Retraction: Analysis of the Prevalence, Secretion and Function of a Cell Cycle-Inhibiting Factor in the Melioidosis Pathogen *Burkholderia pseudomallei*

**DOI:** 10.1371/journal.pone.0283920

**Published:** 2023-03-29

**Authors:** The PLOS ONE Editors

Following the publication of this article [1], concerns were raised regarding results presented in Figs 1 and 4. Specifically,

- The Fig 1A Bacterial lysate K96243 and Bacterial lysate *chbP* results appear more similar than would be expected from independent samples.
- The Fig 4A 6h U937+*chbP*/pCHBP and 6h U937+*bsaQ* results appear to be more similar than would be expected from independent samples.
- There appear to be repetitive patterns in the Fig 4A results.

The corresponding author disagreed that the Figs 1A and 4A results listed above appear more similar than expected and commented that the protein profile of the *Burkholderia pseudomallei* strain K96243 lysate and its isogenic *chbP* mutant in Fig 1A, as well as the lysates of the U937 cells in Fig 6A, are expected to look similar, as there is only a minor difference in predicted proteins. Underlying data were provided for the Fig 1A Bacterial lysate and Secreted protein panels. The underlying data provided for the Bacterial lysate results suggested that the panel was spliced during figure preparation, and the Bacterial Lysate K96243 lane did not appear to match the underlying data provided. The underlying data provided for the Secreted protein results showed that the results were not aligned to the marker lane appropriately; the series of bands aligned with the 95kDa marker in the published results instead appeared just below the 72kDa marker in the underlying data, and the series of bands aligned with just below the 17kDa marker in the published results instead appeared in line with the 10kDa marker in the underlying data.

The corresponding author was unable to explain the appearance of the repetitive features in the Fig 4A results, but they stated that there are also differences between the Fig 4A results listed above. The underlying data provided for the Fig 4A results did not explain the appearance of the repetitive features either, but the data provided appear to confirm the published results. However, upon examination of the underlying data provided for the Fig 4B results, additional concerns were raised that in the underlying data provided the backgrounds for the following lanes appear more similar than would be expected from independent data:

- BopE “U937+K9 3h” and CHBP “Uninfected U937”
- BopE “U937+*chbP* 3h” and CHBP “U397+K9 6h”
- BopE “U937+*chbP/*pCHBP 3h” and CHBP “U397+*chbP* 3h”
- BopE “U937+*bsaQ* 3h” and CHBP “U937+*chbP/*pCHBP 3h”
- BopE “U937+K9 6h” and CHBP “U937+K9 3h”
- BopE “U937+*chbP/*pCHBP 6h” and CHBP “U397+*chbP* 6h”

In light of the concerns affecting multiple figure panels and underlying data that question the integrity of these data, the *PLOS ONE* Editors retract this article.

VM, MPS, and SK agreed with the retraction. PP and NJ did not agree with the retraction and stand by the article’s findings. SK apologizes for the issues with the published article. CVB, PK, KP, and JMS either did not respond directly or could not be reached.
